# The Effect of Frying Conditions on the Physical and Chemical Quality Attributes of Clearhead Icefish (*Protosalanx hyalocranius*) During Deep Frying and Air Frying

**DOI:** 10.3390/foods14060920

**Published:** 2025-03-08

**Authors:** Ying Li, Xiufang Xia, Guoping Yu

**Affiliations:** 1 College of Food Science, Northeast Agricultural University, Harbin 150030, China; liying_and@163.com; 2 School of Food Engineering, East University of Heilongiiang, Harbin 150066, China

**Keywords:** fried clearhead icefish, deep frying, air frying, frying conditions, quality attributes, maillard reaction, mass transfer, texture analysis

## Abstract

The effect of frying conditions on the quality attributes of clearhead icefish under deep frying and air frying was evaluated using the Soxhlet extractor method, colorimeter, and textural analyzer. With the increasing frying temperature and time, the water loss, oil uptake, volumetric shrinkage, redness, yellowness, browning index, hardness, crispiness, the dispersion of textural data, and sensory scores in deep-fried and air-fried samples increased (*p* < 0.05); the lightness, whiteness, and thiobarbituric acid reactive substances (TBARS) decreased (*p* < 0.05), while crispiness showed no significant difference (*p* > 0.05). Compared to deep-fried samples, air-fried samples showed a 15.6–20.8% and 63.2–64.7% decrease in the water content and oil content, respectively. Volumetric shrinkage, hardness, and crispiness of the air-fried samples increased 30.3–68.4%, 53.5–53.7%, and 53.0–59.1%, respectively, relative to deep-fried samples. Air-fried samples displayed a decreasing uniformity in color. Therefore, frying temperature and time conferred a desirable color and texture to the fried clearhead icefish by affecting mass transfer, Maillard reactions, and lipid oxidation reactions. In addition, the difference in quality attributes between deep-fried and air-fried products was attributed to the difference in heat transfer mode. The study aims to provide a theoretical basis for the application of two frying methods and the production of high-quality fried foods.

## 1. Introduction

Clearhead icefish (*Protosalanx hyalocranius*) is a kind of freshwater fish commonly found in Northeast China, known for its small size, transparent body, and lack of bones and guts. Moreover, clearhead icefish are rich in protein, amino acids, and other trace elements, with high nutritional value. Frying is one of the most suitable processing methods for clearhead icefish. Fried clearhead icefish has become a popular fried food because of its easy preparation, golden color, and crispy texture. Deep frying is the most traditional thermal processing method, which uses edible oil as the heat transfer medium, and the high temperature makes the water evaporate rapidly, resulting in the oil entering the water vapor channel and forming a crispy surface layer [1]. In addition, the proteins and amino acids of clearhead icefish and the reduction in sugar at high temperatures causes the Maillard reaction, which gives the product an attractive color. However, deep-fried food is prone to oxidative rancidity and high oil content, which increases the risk of obesity, hypertension, and cardiovascular diseases [2]. Recently, several novel frying technologies including electric field frying [3], vacuum frying [4], and air frying [5] have overcome the defects of traditional frying and are expected to replace deep frying, which has become the direction of the researchers’ efforts. Yang et al. [3] reported that novel electric field frying improved water retention and decreased the oil content of fried shrimp. Chu et al. [4] found that compared to conventional frying, air frying reduced the oil absorption of oysters, and vacuum frying better preserved the flavor of oysters and reduced lipid oxidation. Among these methods, air frying has attracted great interest from health-conscious consumers due to its oil-free, low-fat, and low-calorie characteristics [6]. Air frying operates by circulating the hot air at high speed in a closed space, thus dehydrating the food and making the surface golden and crispy [5]. Compared to deep frying, air frying is more convenient, safer, and easier to operate, which makes it popular at household and fast-food restaurants.

During frying, the quality of fried clearhead icefish is influenced by several factors, including pre-treatment methods [7,8], process parameters [9], and oil type [10]. Temperature and time are two important factors affecting the quality of fried foods, thus controlling frying conditions is fundamental to achieving high-quality fried products. Qin et al. [11] demonstrated that frying contributes to the development of a golden color in food; however, as frying time increases, the surface color transitions from golden to a darker brown. Chu et al. [4] observed that low-temperature, prolonged frying leads to the hardening of the fish skin surface and increase in fracture force. Consequently, selecting appropriate frying methods and conditions is crucial for producing high-quality fried foods. However, there are few studies comparing the effects of deep frying and air frying on the quality attributes of products.

Therefore, this study aimed to (1) investigate the influence of frying conditions on mass transfer, volumetric shrinkage, appearance, color, textural properties, and lipid oxidation under deep frying and air frying; and (2) establish correlations between quality attributes and analyze the underlying causes of quality changes.

## 2. Materials and Methods

### 2.1. Materials and Regents

Fresh dressed clearhead icefish were purchased from the local market. All other chemicals including petroleum ether, trichloroacetic acid, and thiobarbituric acid were of analytical grade and obtained from Tianjin Tianli Chemical Co., Ltd. (Tianjin, China).

### 2.2. Preparation of Frying Clearhead Icefish

**Figure 1:**
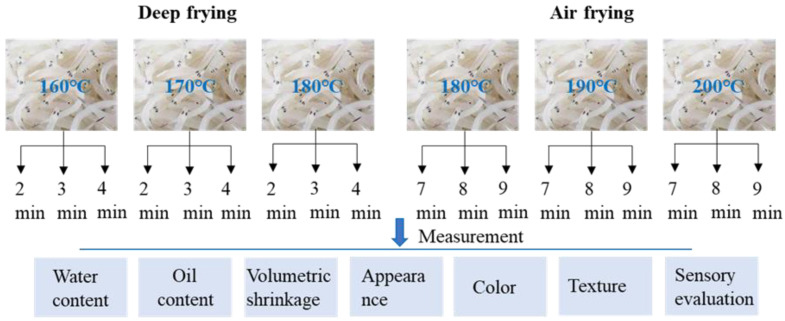
Experimental design diagram.

Clearhead icefish are elongated and have a body length of approximately 10 cm. Clearhead icefish of uniform size were selected, cleaned, drained, and weighed. Following this, the fish was marinated with 1.33% (*w*/*w*) sugar, 2.0% (*w*/*w*) salt, and cooking wine for 30 min. After marination, the clearhead icefish were pre-dried in an electric thermostatic drying oven (DHG-9013A, Shanghai, China) at 60 °C for 3 h. A total of 1 L of fresh soybean oil was added to a temperature-controlled fryer (AS-168, Ningbo, China). The range of frying temperature and time for deep frying and air frying was determined by the sensory characteristics of the pretested samples. One portion of the pre-dried clearhead icefish was submerged in the frying oil and deep-fried at 160 °C, 170 °C, and 180 °C for 2 min, 3 min, and 4 min, respectively. Fresh soybean oil was used each time. The remaining pre-dried fish was placed into the cavity chamber of an air fryer (CZG-D05, Jinhua, China), and the frying temperatures were set at 180 °C, 190 °C, and 200 °C for 7 min, 8 min, and 9 min. Hot air was circulated at high speed in the confined space to dehydrate the food. Fish mass relative to fryer capacity per deep and air frying process was 10 g/L and 25 g/L, respectively. The control was raw samples. The samples obtained from the deep frying and air frying were collected for the subsequent analysis and evaluation (Figure 1).

### 2.3. Water Content

The water content of the samples was assessed following GB 5009.3-2016 [12]. Approximately 5–6 g of chopped samples was weighed and dried in an electric thermostatic drying oven (FYD-GF-9053A, Shanghai, China) at 105 °C until a constant weight was reached. The water content was expressed as g/100 g.

### 2.4. Oil Content

The oil content of samples subjected to deep frying and air frying was measured according to GB 5009.6-2016 [13]. After water removal, samples (2–5 g) were transferred into a filter paper cartridge. The filter paper cartridge was placed into the extraction cylinder of a cable extractor and extracted using petroleum ether (30–60 °C) in a water bath at 50 °C for 6–8 h. After extraction, the solvent in the receiver bottle was evaporated and dried in an electric thermostatic drying oven (FYD-GF-9053A, Shanghai, China).

### 2.5. Volumetric Shrinkage

The volumetric shrinkage of the samples after frying was assessed using the millet replacement method [14]. The volumetric shrinkage was calculated by Equation (1):

(1) $$Volumetric\;shrinkage\;\left(\%\right)=\frac{V_{0}-V_{f}}{V_{0}}\times 100$$

where *V*_0_ is the volume of the raw sample, and *V_f_* is the volume of the samples fried at different frying temperatures and times under deep frying and air frying.

### 2.6. Color

The color parameters of fresh and fried clearhead icefish were determined using a ZE-6000 colorimeter (Juki Corp., Tokyo, Japan). The whiteness (*W*) and browning index (*BI*) values of the samples were obtained by Equations (2)–(4) [15,16]:

(2) $$W=100-\sqrt{{\left(100-L^{*}\right)}^{2}+{a^{*}}^{2}+{b^{*}}^{2}}$$

(3) $$BI=(100\times \left(x-0.31\right))/0.17$$

(4) $$x=(a^{*}+1.75L^{*})/(5.645L^{*}+{a_{0}}^{*}-3.012b^{*})$$

where *a*_0_*: the color of the raw samples; lightness (L*), redness (a*), yellowness (b*): the color of deep-fried and air-fried samples at different temperatures and times.

### 2.7. Texture

The textural parameters of the samples were determined by the method of Bhuiyan and Ngadi [17]. After frying, the surface temperature of the samples was allowed to cool to 50 °C for the determination of the texture. A P/2 probe (2 mm diameter) of a texture analyzer (TA.XT PlusC, Stable Micro Systems Ltd., Godalming, UK) was used for the puncture test on the samples (center). The pre-test and test speeds were 2 mm/s, and the post-test speed was 10 mm/s. The test was repeated nine times, and the force-time curves and textural parameters of the samples were obtained.

### 2.8. Thiobarbituric Reactive Substances (TBARS)

The TBARS values of fried clearhead icefish were evaluated by the method of Chen et al. [18]. Clearhead icefish (5 g) was mixed with 25 mL of 7.5% (*v*/*v*) trichloroacetic acid. The mixture was shaken for 30 min and filtered, then 5 mL of 0.02 mol/L thiobarbituric acid was added to the filtrate. The mixture was placed in a boiling water bath for 40 min, cooled, and centrifuged (6000 rpm, 4 °C) for 25 min. The absorbance of the supernatant at 532 nm (A_532_) and 600 nm (A_600_) was recorded. The TBARS values were calculated by Equation (5):

(5) $$\mathrm{TBARS}\;(\mathrm{mg}/\mathrm{kg})=\frac{A_{532}-A_{600}}{155}\times 72.06\;\times \frac{1}{10}\times 1000$$

### 2.9. Sensory Evaluation

The fried clearhead icefish were placed in plastic boxes and offered to 20 trained panelists. The panelists evaluated four sensory attributes: color, texture, flavor, and overall acceptability (on a scale of 1–9). The sensory evaluation was carried out under the approval permission of the Ethics in Research Committee of Northeast Agricultural University (NEAUEC202404122). Ratification date was 28 November 2024. All the panelists agreed voluntarily to participate in the sensory evaluation.

### 2.10. Statistical Analysis

All experiments were performed in three replicates. The results were expressed as mean values ± standard error using Statistic 8.1 software (Analytical Software, Tallahassee, FL, USA), and analysis of variance (ANOVA) with Tukey’s multiple comparisons was performed to compare the data at a significance level of *p* < 0.05 [19]. Data regarding the quality attributes of fried samples were analyzed using a mixed model according to the analytical methods of Zhao et al. [20]. In this model, the fixed terms included frying temperature and time, and each replication was included as a random term. Frying temperature and time are independent variables. Factor levels include 160 °C, 170 °C, and 180 °C and 180 °C, 190 °C, and 200 °C at 2 min, 3 min, and 4 min and 7 min, 8 min, and 9 min. Graphs were provided using Origin 2024 (Cambridge, MA, USA).

## 3. Results and Discussion

### 3.1. Water and Oil Content

The effect of the frying conditions on the water and oil content of the samples subjected to deep frying and air frying is shown in Table 1 and Table 2. The water content of the raw clearhead icefish was 80.8% (*w*/*w*). With increasing temperature and time, the water content of all fried samples under deep frying and air frying showed a significant decrease (*p* < 0.05). This was attributed to the heat transferred from the frying medium to the surface of products by convection heat transfer and then by conduction heat transfer to the interior of products, resulting in more water evaporation. Compared to the control, the water content of clearhead icefish fried at 160 °C, 170 °C, and 180 °C for 4 min under deep frying decreased by 72.5%, 80.8%, and 86.2%; the samples fried at 180 °C, 190 °C, and 200 °C for 9 min under air frying was reduced by 63.7%, 70.5%, and 83.4%. This indicated that the moisture of clearhead icefish evaporated rapidly in a short period during deep frying, while air frying required higher temperature and a longer time to obtain a lower moisture content. It was related to the differences in heat transfer between deep frying and air frying [21]. The samples were in direct contact with the oil in the deep-frying process, whereas the heat was transferred through the external emulsion of the oil droplets in the hot air during air frying, causing a slower heat transfer [22]. Fang, Huang, and Sung [23] also found a slower decrease in the moisture content of the fish skin by air frying compared to deep frying.

The oil content of unfried samples was 4.6% (*w*/*w* dry basis). A significant increase in oil content could be observed in all samples by deep and air frying compared to the unfried samples, which was associated with the formation of pores by moisture migration during frying, providing space for oil penetration. The oil content of deep-fried samples ranged from 17.9% to 25.1%. With the increasing deep-frying time, there was no significant change in the oil content of samples fried at 160 °C and 170 °C, while it decreased significantly at 180 °C. With the increasing deep-frying temperature, the oil content of the samples fried for 2 min and 3 min increased, while the samples fried for 4 min showed no significant difference. It was explained by the dramatic evaporation of water at higher temperatures, which formed dense bubbles on the surface, making it difficult for the oil to enter the food [24]. The oil content of air-fried samples ranged from 6.0 to 9.2%. For air-fried products, the oil content increased significantly with increased frying temperature and time. This was attributed to an increase in the temperature and number of hot air cycles, which led to an increase in the pore size and number of water vapor channels formed inside the products, facilitating oil penetration [25]. The leaching of fish fat also resulted in more oil content. Compared to deep-fried samples, air-fried samples showed a decrease in oil content of 63.2–64.7%, which was because the products were immersed in the oil during frying.

### 3.2. Volumetric Shrinkage

**Figure 2:**
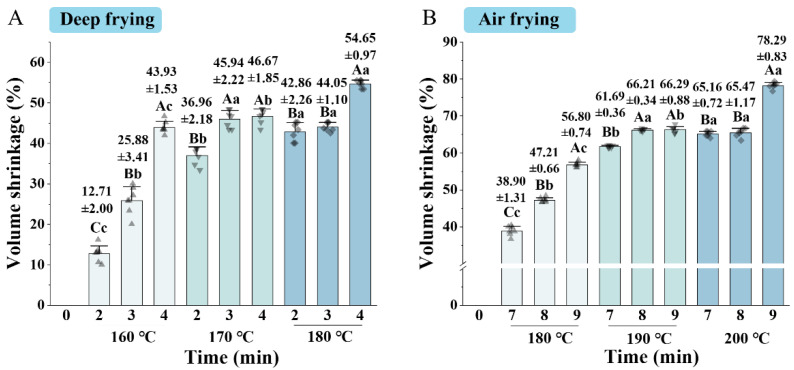
Effect of frying conditions on the volumetric shrinkage of clearhead icefish (*Protosalanx hyalocranius*) fried under different frying methods. (**A**) volume shrinkage of deep-fried samples; (**B**) volume shrinkage of air-fried samples. Different uppercase letters (A–C) indicate significant differences within different time (*p* < 0.05), and different lower-case letters (a–c) indicate significant differences within different temperature (*p* < 0.05).

The volumetric shrinkage can reflect the mass transfer and structural changes in the samples during frying. The effects of frying methods and parameters on the volumetric shrinkage of clearhead icefish are exhibited in Figure 2. With the increasing frying temperature and time, the volumetric shrinkage of all samples subjected to deep frying and air frying increased significantly (*p* < 0.05). It was associated with water evaporation and contraction of muscle fibers [26]. At high temperatures, structures and cell walls in food products become fragile, and the internal and external pressure difference caused by uneven water evaporation may lead to cell rupture or collapse. Li et al. [14] also described that volumetric shrinkage was dominated by water loss and oil uptake. The volumetric shrinkage of deep-fried and air-fried samples ranged from 12.7 to 54.6% and 38.9–78.3%, respectively. The volumetric shrinkage of deep-fried samples was significantly lower than that of air frying. This was attributed to the difference in heat transfer mechanisms between the two frying methods. During deep frying, edible oil acts as the heat transfer medium, and water evaporation and oil absorption occur almost simultaneously. Several pores and cracks formed with water evaporation were filled by oil, which acted as a structural support, reducing the volumetric shrinkage [27]. In the air frying process, the high-speed circulation of hot air causes the rapid dehydration of the product, increasing in volumetric shrinkage [4]. Moreover, the longer mass transfer process during air frying was also responsible for the volumetric shrinkage.

### 3.3. Color

**Figure 3:**
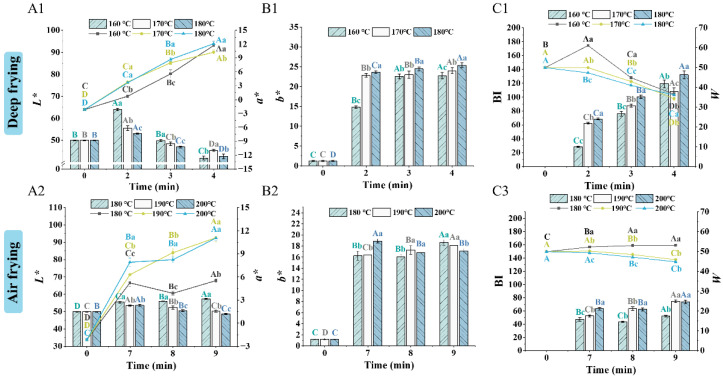
Effect of frying conditions on the color properties of clearhead icefish (*Protosalanx hyalocranius*) fried under different frying methods. (**A1**): L* and a* of deep-fried samples; (**B1**): b* of deep-fried samples; (**C1**): *BI* and *W* of deep-fried samples. (**A2**): L* and a* of air-fried samples; (**B2**): b* of air-fried samples; (**C2**): *BI* and *W* of air-fried samples. Different uppercase letters (A–D) indicate significant differences within different time (*p* < 0.05), and different lower-case letters (a–c) indicate significant differences within different temperature (*p* < 0.05).

The color of fried products is a key quality attribute that attracts consumers. The color of fried foods is measured using a colorimeter; higher lightness (L*) and yellowness (b*), and lower redness (a*), are generally desirable for fried foods. The color properties of fried clearhead icefish are shown in Figure 3. BI values reflect the browning degree of the products. Compared to the raw samples, the L* values of samples fried at 160 °C under deep frying increased, while the values for other samples decreased. The a* and b* of all samples increased while the *W* values decreased. As the frying temperature and time increased, the L* and *W* values of samples subjected to deep frying decreased while a*, b*, and *BI* increased. This phenomenon was attributed to non-enzymatic browning reactions, namely, the Maillard reaction and caramelization. The Maillard reaction uses carbonyl and amino compounds as precursors and is divided into three stages. The advanced stage is key in the formation of colored substances. This stage consists of a series of reactions in which the chemically active end-products from the intermediate stages undergo oxidation, dehydration, cyclization, rearrangement, and condensation, and ultimately produce brown-pigmented substances and melanoidins [28]. Caramelization is the process of dehydrating and degrading the saccharides, especially the monosaccharides, by heating above 140–170 °C in the absence of amino compounds. The degradation products of saccharides are of mainly two types: (1) the products of aldose or ketose generated by enolization and dehydration under acidic conditions (i.e., caramel color), and (2) cleavage products such as volatile aldehydes and ketones, which are further condensed and polymerized to produce black pigments, resulting in browning [29,30].

For the air-fried samples, with increasing frying temperature and time, the L*, a*, b*, *W*, and *BI* values showed similar trends to those of deep-fried samples. Higher frying temperatures and times had lower b* values. Compared to deep-fried samples, the a*, b*, and *BI* values decreased, and the *W* values increased, which indicated a lower degree and rate of the non-enzymatic browning reactions. This phenomenon was related to the increase in humidity inside the chamber caused by less water loss during air frying [5]. Therefore, the deep-fried samples exhibited a color that was more likely to be selected by consumers.

### 3.4. Texture

**Figure 4:**
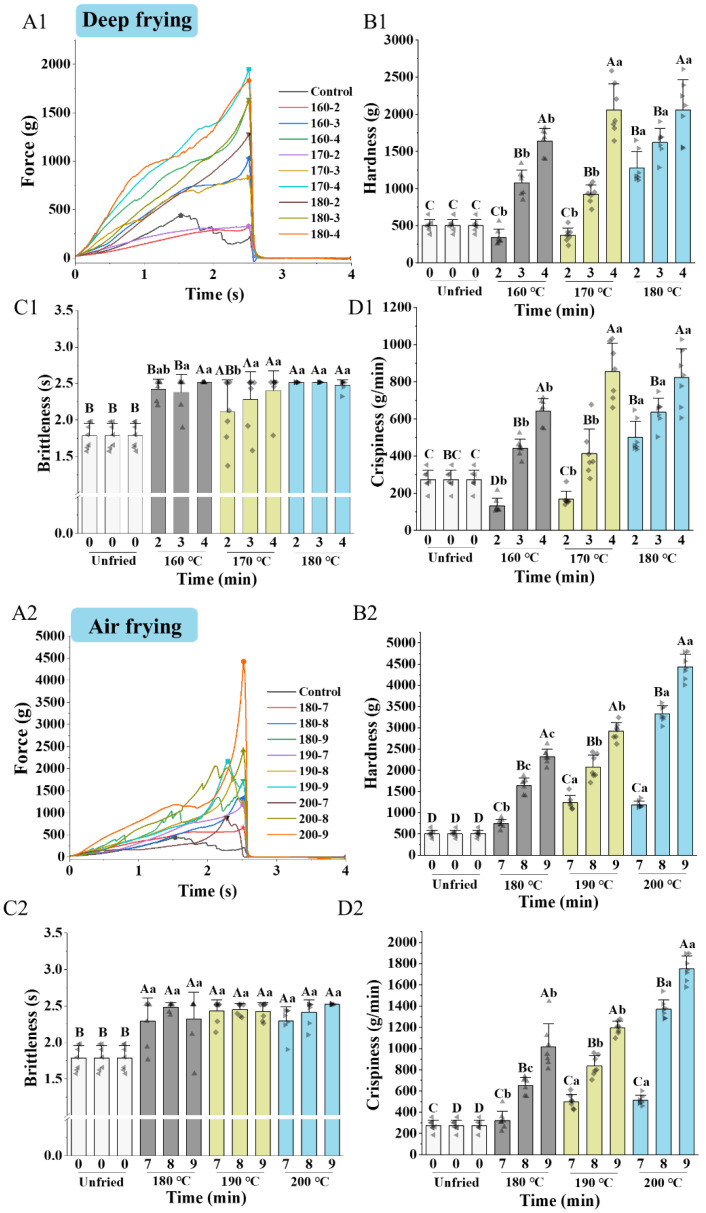
Effect of frying conditions on the textural properties of clearhead icefish (*Protosalanx hyalocranius*) fried under different frying methods. (**A1**–**D1**): time-force curves, hardness, brittleness, crispiness of deep-fried samples, respectively; (**A2**–**D2**): time-force curves, hardness, brittleness, crispiness of air-fried samples, respectively. Different uppercase letters (A–D) indicate significant differences within different times (*p* < 0.05), and different lower-case letters (a–c) indicate significant differences within different temperatures (*p* < 0.05).

Brittleness and crispiness are the key factors in the popularity of fried fish among consumers. The force-time curves and textural parameters obtained from the puncture test are exhibited in Figure 4. The force-time curves of all samples showed an increase followed by a decrease. The maximum force, time at maximum force, and slope at maximum force on the curves represented the hardness, brittleness, and crispiness of the samples, respectively. Deep frying and air frying increased the hardness and crispiness of clearhead icefish. Sanz, Primo-Martin, and Vliet [31] reported that the number of maximum force events and sound events increased after frying, indicating an enhanced crust crispness of fried meat.

As the deep-frying temperature and time increased, the hardness and crispiness of the samples significantly increased (*p* < 0.05). The brittleness of samples deep-fried at 160 °C decreased with time, while other samples showed no significant difference. For air frying, with increasing frying temperature and time, the hardness and crispiness of the samples increased significantly. There was no significant difference in the crispiness of all air-fried samples (*p* > 0.05). Li et al. [27] found that the shear force of fried chicken gizzards increased as the frying time prolonged, which was attributed to myofibrillar contraction and water loss. During frying, the difference in vapor pressure between the inner and outer surface of the food forces water to escape, leading to the creation, growth, and fusion of pores and increasing pore size and crispness [32]. Moreover, heat transfer and water loss led to the thermal contraction of muscle fibers and protein denaturation and aggregation, which increased the hardness of the samples [33].

The hardness and crispiness of the air-fried samples were higher than deep-fried samples, which was associated with more moisture loss and thermal contractions of muscle fibers in air frying [34]. The data points for textural parameters were more discretely distributed with increasing temperature and time for both deep frying and air frying, which suggested a larger heterogeneity in the texture of a batch of samples from the same treatment. These phenomena were due to differences in heat and mass transfer between the surface and interior of the samples.

### 3.5. TBARS

**Figure 5:**
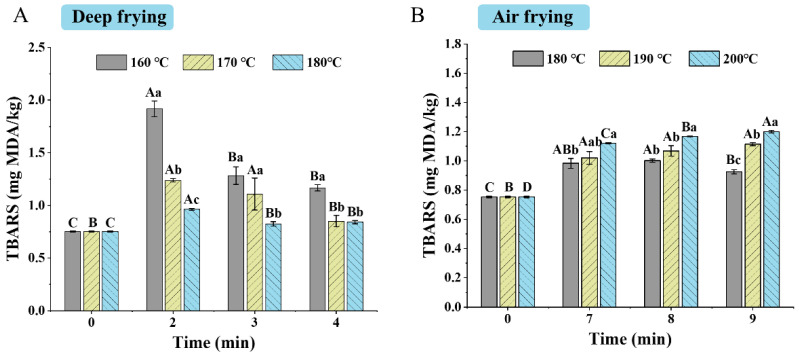
Effect of frying conditions on the TBARS values of clearhead icefish (*Protosalanx hy-alocranius*) fried under different frying methods. (**A**): TBARS of deep-fried samples; (**B**): TBARS of air-fried samples. Different uppercase letters (A–D) indicate significant differences within different time (*p* < 0.05), and different lower-case letters (a–c) indicate significant differences within different temperature (*p* < 0.05).

The effect of frying conditions on TBARS of the samples under different frying methods is shown in Figure 5. The TBARS value of raw clearhead icefish was 0.75 mg MDA/kg, and the frying treatment increased the TBARS values of the samples. The TBARS values ranged from 1.17 to 1.92 mg/kg, 0.85–1.24 mg/kg, and 0.82–0.96 mg/kg for the deep-fried samples at 160 °C, 170 °C, and 180 °C, respectively. With the rise in frying temperature and time, the TBARS values of deep-fried samples decreased. It might be due to the reaction of several aldehydes with proteins, amino acids, and other substances containing primary ammonia under higher temperatures and longer frying times. Qin et al. [11] also found with the frying time prolonged, the lipid oxidation of fried fish nuggets decreased.

The TBARS values of air-fried samples ranged from 0.92 to 1.00 mg/kg, 1.02–1.11 mg/kg, and 1.12–1.19 mg/kg at 180 °C, 190 °C, and 200 °C. With the increasing temperature, the TBARS values of the air-fried samples increased. With the increasing time, the TBARS values of samples air-fried at 180 °C decreased, while samples fried at 190 °C and 200 °C showed an increase in TBARS values. It indicated an increase in the lipid oxidation of the samples at higher temperatures and for longer periods. Compared to deep frying, samples air-fried for 2 min and 3 min had lower TBARS values, while samples air-fried for 4 min had higher TBARS values. This was caused by the continuous heating cycle of hot air [35]. The reduced lipid oxidation was more noticeable in deep frying than in air frying. A similar phenomenon was found by Qin et al. [11] who explained that as the frying time was extended from 3 min to 8 min, the TBARS values of deep-fried fish nuggets decreased, whereas the TBARS values of air-fried samples increased.

### 3.6. Appearance and Sensory Evaluation

**Figure 6:**
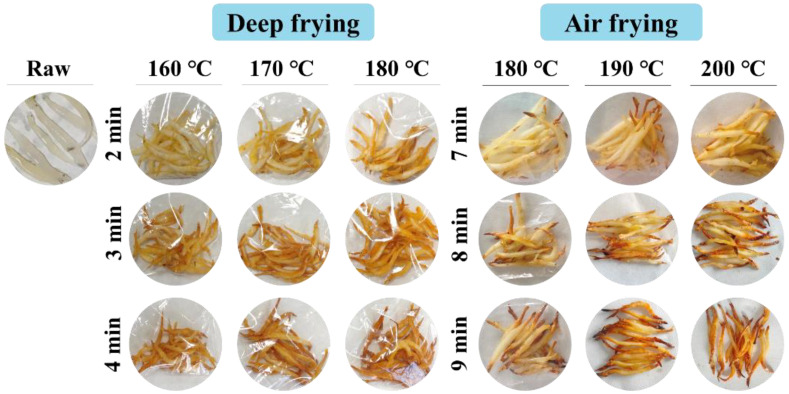
Effect of frying conditions on the appearance of clearhead icefish (*Protosalanx hyalocranius*) fried under different frying methods.

The appearance of clearhead icefish is exhibited in Figure 6. The raw samples were white and translucent with a smooth surface. At the early stage of deep frying, the edges, head, and tail areas of the samples were light yellow. As the frying proceeded, the light-yellow area increased, the color became darker, and the surface was relatively smooth. At higher temperatures and times, the samples were brownish-yellow with burnt edges, and the surface became rough with irregular protrusions and pores visible to the naked eye. This was caused by the increase in Maillard reaction and water migration [36]. The appearance of samples fried at 180 °C, 190 °C, and 200 °C for 7 min showed a charred head and tail and white center, and the surface was smooth. With the extension of time, the charred area increased, the middle area gradually became light yellow, and the irregular protrusions at the head and tail increased. Compared to the deep-fried samples, the uniformity of the color of the air-fried samples was poor, but the greasiness was reduced.

The changes in appearance can be scientifically analyzed by the results of sensory evaluation (Table 3). When the deep-frying temperature was 160 °C, the scores for color, texture, flavor, and overall acceptability of the products increased significantly with increasing time (*p* < 0.05). At deep-frying temperatures of 170 °C and 180 °C, the scores for color, texture, flavor, and overall acceptability increased and then decreased. For the air-frying samples, the sensory scores increased significantly at 180 °C, and the sensory scores increased and then decreased at 190 and 200 °C. These phenomena were attributed to the undesirable color and texture caused by excessive heat and mass transfer and the Maillard reaction.

### 3.7. Correlation Analysis

**Figure 7:**
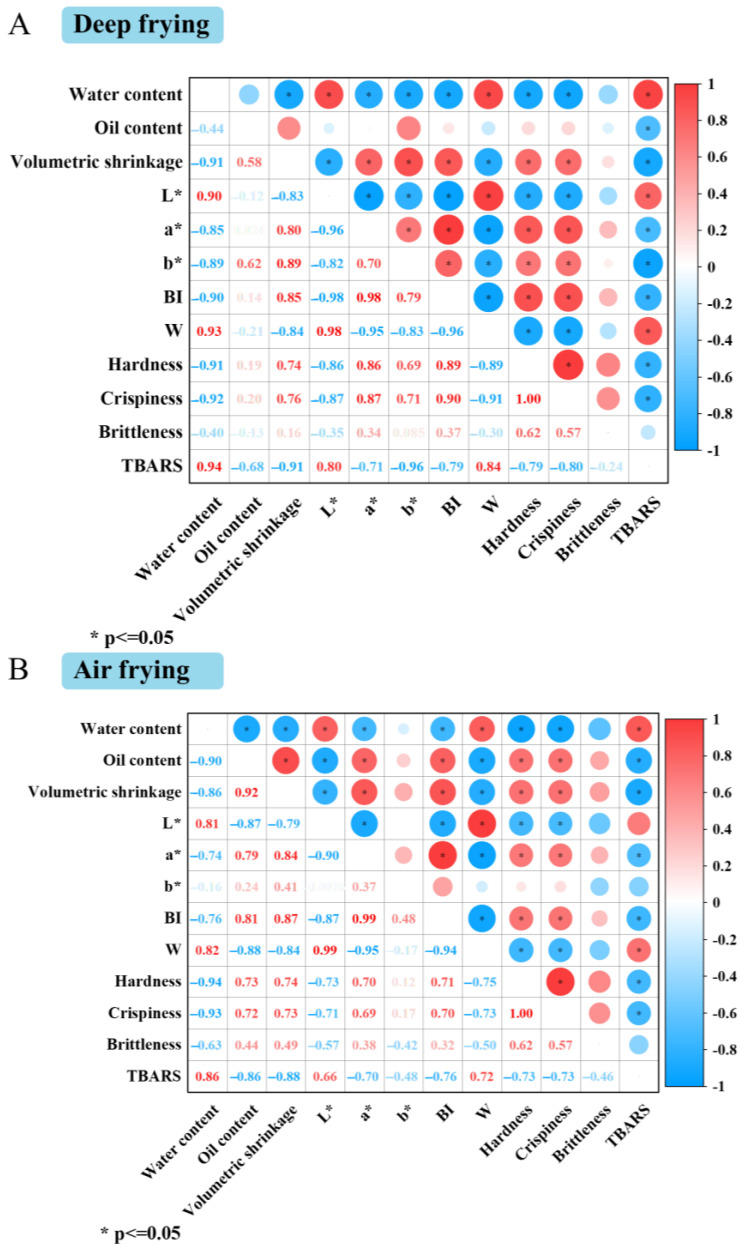
Correlation analysis of quality attributes of fried clearhead icefish under deep frying (**A**) and air frying (**B**).

The correlation analysis between the quality attributes of deep-fried and air-fried samples is shown in Figure 7. The water content of deep-fried samples showed a significant positive correlation with L*, *W*, and TBARS values and a significant negative correlation with volumetric shrinkage, a*, b*, *BI*, hardness, crispiness, and brittleness (*p* < 0.05). A similar phenomenon was observed in the correlation of these quality attributes in the air-fried samples. Due to this, the water migration promoted the non-enzymatic browning reaction and the formation of porous structures during frying, leading to increased browning degree and crispiness of the samples [36]. In addition, there was a dry and hard crust on the surface of the sample in the early stage of frying. As the frying proceeded, the internal moisture continued to migrate driven by the difference in the internal and external water vapor pressure, which increased the crust thickness, leading to an increase in the hardness and volumetric shrinkage of the samples [37,38]. The oil content of deep-fried samples was positively correlated with the b* and volumetric shrinkage. The increasing b* values with increasing oil content were related to the color of the frying oil itself. Moreover, the entry of oil acted as a support structure to prevent cell collapse due to water evaporation, thus reducing the volumetric shrinkage [39].

There was a strong negative correlation between the moisture content and oil content of the air-fried samples with a correlation coefficient of −0.86 (*p* < 0.05), which was higher than the correlation coefficient between the two for deep-fried samples. It was explained that water evaporation was more intense during deep frying, increasing the force the oil penetrated to the interior of the samples [40].

## 4. Conclusions

In this study, the effect of the frying conditions on the quality attributes of clearhead icefish under different methods was evaluated. With the increase in frying temperature and time, the mass transfer (water loss and oil absorption) and Maillard reaction of deep-fried and air-fried samples increased, forming a porous support structure, which imparted a golden color and crispy texture to clearhead icefish. However, higher temperatures and longer time resulted in darkening of color, formation of dry and hard texture, and uneven product quality. The samples deep-fried at 160 °C for 4 min and air-fried at 180 °C for 9 min were more satisfactory. The effect of frying temperature on the dependent variables did not depend on the frying time, thus there is no interaction between frying temperature and frying time. The variability in the quality of the samples under different frying methods was greater. Compared to deep-fried samples, air-fried samples absorbed less oil, which is conducive to reducing the incidence of many chronic diseases such as cardiovascular disease and obesity. However, the air-fried samples showed uneven color and increased hardness, which need to be further improved. This study provides a theoretical basis for the development of novel frying methods and the industrial application of low-oil fried foods. Expanding the industrial application of air frying and improving the quality of products is the focus of future research.

## Figures and Tables

**Table 1:** Effect of frying conditions on the water content of clearhead icefish (*Protosalanx hyalocranius*) under different frying methods.

Frying Methods	Frying Parameters											
Deep frying	160 °C				170 °C				180 °C			
	0 min	2 min	3 min	4 min	0 min	2 min	3 min	4 min	0 min	2 min	3 min	4 min
	80.79 ± 0.54 ^A^	48.77 ± 2.35 ^B,a^	28.48 ± 2.06 ^C,a^	22.75 ± 0.36 ^D,a^	80.79 ± 0.54 ^A^	36.27 ± 0.26 ^B,b^	22.56 ± 1.36 ^C,b^	15.50 ± 0.64 ^D,b^	80.79 ± 0.54 ^A^	22.53 ± 1.04 ^B,c^	18.47 ± 0.71 ^C,c^	11.17 ± 1.24 ^D,c^
Air frying	180 °C				190 °C				200 °C			
	0 min	7 min	8 min	9 min	0 min	7 min	8 min	9 min	0 min	7 min	8 min	9 min
	80.79 ± 0.54 ^A^	38.42 ± 0.25 ^B,a^	29.90 ± 0.26 ^C,a^	29.34 ± 0.67 ^C,a^	80.79 ± 0.54 ^A^	30.82 ± 1.24 ^B,b^	26.97 ± 0.71 ^C,b^	23.85 ± 1.15 ^D,b^	80.79 ± 0.54 ^A^	28.21 ± 0.71 ^B,c^	18.12 ± 0.59 ^C,c^	13.41 ± 0.31 ^D,c^

Different uppercase letters (A–D) indicate significant differences within different time (*p* < 0.05), and different lower-case letters (a–c) indicate significant differences within different temperature (*p* < 0.05).

**Table 2:** Effect of frying conditions on the oil content of clearhead icefish (*Protosalanx hyalocranius*) under different frying methods.

Frying Methods	Frying Parameters											
Deep frying	160 °C				170 °C				180 °C			
	0 min	2 min	3 min	4 min	0 min	2 min	3 min	4 min	0 min	2 min	3 min	4 min
	4.63 ± 0.20 ^B^	17.92 ± 1.05 ^A,b^	19.05 ± 1.09 ^A,b^	18.57 ± 1.85 ^A,a^	4.63 ± 0.20 ^B^	23.64 ± 0.33 ^A,a^	21.77 ± 0.88 ^A,a^	22.35 ± 1.66 ^A,a^	4.63 ± 0.20 ^B^	25.05 ± 0.93 ^A,a^	23.50 ± 0.55 ^A,B,a^	21.93 ± 1.13 ^B,a^
Air frying	180 °C				190 °C				200 °C			
	0 min	7 min	8 min	9 min	0 min	7 min	8 min	9 min	0 min	7 min	8 min	9 min
	4.63 ± 0.20 ^C^	6.02 ± 0.46 ^B,b^	6.55 ± 0.62 ^B,b^	6.77 ± 0.35 ^A,a^	4.63 ± 0.20 ^B^	7.64 ± 0.34 ^A,a^	7.77 ± 0.48 ^A,a^	7.95 ± 0.36 ^A,a^	4.63 ± 0.20 ^C^	8.25 ± 0.73 ^B,a^	9.00 ± 0.15 ^A,B,a^	9.21 ± 0.14 ^A,a^

Different uppercase letters (A–C) indicate significant differences within different time (*p* < 0.05), and different lower-case letters (a–b) indicate significant differences within different temperature (*p* < 0.05).

**Table 3:** Effect of frying conditions on the sensory evaluation of clearhead icefish (*Protosalanx hyalocranius*) under different frying methods. A–C: xxxx; a,b: xxxx.

Frying Methods	Temperature (°C)	Time (min)	Color	Texture	Flavor	Overall Acceptability
Deep frying	160	2	5.26 ± 0.45 ^B,a^	4.27 ± 0.35 ^C,b^	7.21 ± 0.54 ^A,a^	6.53 ± 0.75 ^B,b^
		3	5.63 ± 0.64 ^B,b^	5.98 ± 0.65 ^B,b^	7.54 ± 0.29 ^A,a^	6.55 ± 0.45 ^B,b^
		4	7.93 ± 0.53 ^A,a^	7.83 ± 0.66 ^A,a^	8.04 ± 0.83 ^A,a^	8.56 ± 0.36 ^A,a^
	170	2	5.33 ± 0.62 ^B,a^	5.04 ± 0.64 ^B,a,b^	7.45 ± 0.68 ^A,a^	6.93 ± 0.67 ^A,a,b^
		3	7.66 ± 0.51 ^A,a^	7.80 ± 0.75 ^A,a^	8.05 ± 0.58 ^A,a^	7.29 ± 0.63 ^A,a^
		4	7.10 ± 0.71 ^A,a,b^	7.21 ± 0.54 ^A,a^	7.65 ± 0.74 ^A,a^	7.45 ± 0.75 ^A,a^
	180	2	6.21 ± 0.56 ^A,a^	5.77 ± 0.49 ^A,a^	7.55 ± 0.76 ^A,a^	7.03 ± 0.46 ^A,a^
		3	6.55 ± 0.61 ^A,a,b^	7.02 ± 0.57 ^A,a,b^	8.46 ± 0.73 ^A,a^	7.65 ± 0.65 ^A,a^
		4	6.12 ± 0.55 ^A,b^	6.43 ± 0.64 ^A,a^	8.32 ± 0.46 ^A,a^	6.54 ± 0.16 ^A,a^
Air frying	180	7	4.54 ± 0.45 ^B,a^	4.05 ± 0.43 ^A,a^	6.05 ± 0.37 ^A,a^	5.35 ± 0.65 ^A,b^
		8	4.67 ± 0.32 ^B,a^	4.55 ± 0.54 ^A,a^	6.67 ± 0.64 ^A,a^	5.45 ± 0.68 ^A,b^
		9	6.77 ± 0.59 ^A,a^	5.04 ± 0.64 ^A,a^	6.99 ± 0.69 ^A,a^	5.77 ± 0.47 ^A,a^
	190	7	5.02 ± 0.64 ^A,a^	4.69 ± 0.20 ^A,a^	6.75 ± 0.58 ^A,a^	5.64 ± 0.76 ^A,a,b^
		8	6.02 ± 0.71 ^A,a^	5.95 ± 0.86 ^A,a^	6.88 ± 0.64 ^A,a^	7.43 ± 0.56 ^A,a^
		9	5.79 ± 0.52 ^A,a^	5.99 ± 0.75 ^A,a^	6.87 ± 0.65 ^A,a^	6.54 ± 0.83 ^A,a^
	200	7	5.55 ± 0.65 ^A,a^	5.12 ± 0.65 ^A,a^	6.34 ± 0.68 ^A,a^	7.03 ± 0.54 ^A,a^
		8	5.49 ± 0.68 ^A,a^	5.99 ± 0.65 ^A,a^	6.85 ± 0.65 ^A,a^	7.01 ± 0.54 ^A,a^
		9	6.39 ± 0.57 ^A,a^	6.04 ± 0.59 ^A,a^	6.75 ± 0.57 ^A,a^	6.54 ± 0.73 ^A,a^

Different uppercase letters (A–C) indicate significant differences within different time (*p* < 0.05), and different lower-case letters (a, b) indicate significant differences within different temperature (*p* < 0.05).

## Data Availability

The original contributions presented in the study are included in the article, further inquiries can be directed to the corresponding author.
